# Germline homologous recombination repair (gHRR) variants in bladder cancer: Preliminary evidence and clinical implications

**DOI:** 10.1002/bco2.70077

**Published:** 2025-09-25

**Authors:** Rodolfo Hurle, Anita Capalbo, Giovanni Lughezzani, Nicolò Maria Buffi, Francesco Sormani, Alessio Finocchiaro, Alberto Saita, Marco Paciotti, Vittorio Fasulo, Pietro Cavalli, Paolo Bianchi, Alessio Benetti, Pier Paolo Avolio, Rosanna Asselta, Giulia Soldà, Paolo Casale, Massimo Lazzeri

**Affiliations:** ^1^ Department of Urology IRCCS‐Humanitas Research Hospital Rozzano Italy; ^2^ Department of Biomedical Sciences Humanitas University Pieve Emanuele Italy; ^3^ Medical Genetics and RNA Biology Laboratory IRCCS‐Humanitas Research Hospital Rozzano Italy; ^4^ Laboratory Analysis Unit IRCCS‐Humanitas Research Hospital Rozzano Italy

**Keywords:** bladder cancer, genetic testing, germline variants, homologous recombination repair

## Abstract

**Objective:**

The contribution of germline DNA repair gene (gDRG) variants to bladder cancer (BC) susceptibility and progression is still poorly defined, particularly in European populations. This study aims to evaluate the prevalence and clinical implications of germline homologous recombination repair (HRR) gene variants in BC patients of European ancestry.

**Methods:**

In this prospective case–control study, 75 BC patients attending follow‐up at a single tertiary centre were screened for germline variants in 20 gDRGs. Patients were included regardless of disease stage and classified by pathogenicity (Class 3–5). Clinical characteristics and outcomes were compared between variant‐positive and variant‐negative patients.

**Results:**

Among 75 eligible patients, 72 underwent successful germline sequencing. A total of 23 patients (30.6%) harboured at least one pathogenic, likely pathogenic, or VUS variant. The most frequently altered genes included *ATM* (*n* = 6), *ATR* (*n* = 4), *BARD1* (*n* = 4), *CHEK2* (*n* = 3) and *PMS2* (*n* = 3). Eight patients (34.7%) had multiple variants, and one carried three variants. Notably, 25.8% of NMIBC and 50% of MIBC patients had gDRG variants. Moreover, 30% of patients with low‐grade G1 disease harboured at least one variant. Patients with gDRG variants had a higher rate of histopathological variants (34.8% vs. 13.5%) and underwent radical cystectomy at a younger age (60 vs. 75 years, *p* < 0.05).

**Conclusions:**

Germline HRR variants are prevalent in BC patients and may influence disease aggressiveness and treatment decisions. These findings support broader implementation of germline testing in BC and warrant further validation in larger cohorts.

## 1 INTRODUCTION

According to GLOBOCAN, in 2020, there were 573,000 new cases of bladder cancer (BC).^1^ Age‐standardised incidence rates (ASRs; per 100 000 persons per year) for both sexes combined were highest in Europe (11.0), North America (11.0), North Africa (8.9) and West Asia (8.6), and lowest in West (2.0) and Middle (1.6) Africa, Central America (2.2) and South‐Central Asia (1.9).^2^ In 2020, around 212,000 BC deaths were estimated to occur, representing approximately 3% of all new cancer cases and more than 2% of cancer deaths. BC ranks as the tenth most diagnosed cancer and the 13th most common cause of cancer mortality worldwide. Early conservative treatments or radical cystectomy are effective, showing relatively high survival with 5‐year relative survival rates of around 77% observed in the US and 68% reported in Europe.^1^ GLOBOCAN predictions of future BC disease burden by continent, based on demographic changes only, estimate that the greatest percentage increases in incident BC cases, from 2020 to 2040, will be in Africa (101%), Latin America and the Caribbean (85%), Asia (79%) and Oceania (70%).^11^

Most of BC cases are associated with external risk factors such as tobacco smoking, occupational or environmental exposures.^3^, ^4^ Although BC is rarely attributed to hereditary causes, recent studies suggest inherited factors may play a larger role than previously thought.^5^ Current AUA (American Urological Association), EAU (European Association of Urology) and NCCN (National Comprehensive Cancer Network) guidelines recommend considering genetic evaluation in patients with BC diagnosed at <50 years old or a personal or family history of Lynch Syndrome‐associated cancers. The USPSTF (United States Preventive Services Task Force) does not recommend any BC screening, nor do NCCN, AUA or EAU guidelines.

Genome stability is critical for preventing tumorigenesis, and DNA damage repair systems, involved in the maintenance of genome stability, supply a crucial defence function against DNA‐damaging agents, such as exposure to cigarette smoking, the ultraviolet component of ionizing radiation, or genotoxic substances. Although inherited defects in the DNA repair pathways are known to predispose to various types of malignancies,^6^ the full pathogenic germline variant landscape and optimal genetic evaluation criteria in BC remain poorly understood.^7^ In a large systematic evaluation involving 1038 patients, predominantly of White ethnicity with urothelial cancer, germline class 4/5 variants (pathogenic or likely pathogenic) were identified in 24% of patients, with notably 20% of these variants found in DNA damage repair genes.^8^ In a cohort of 98 Chinese bladder cancer patients, germline DNA damage repair gene variants were detected in 10.2% of individuals.^9^ In a cohort of 206 patients with genitourinary cancers—including 48 cases of bladder urothelial carcinoma—Yang et al. identified pathogenic and likely pathogenic germline variants across multiple genes; notably, ATM and CHEK2 were among the most frequently mutated in bladder cancer.^10^

- Based on the limited literature evidence, there is an unmet clinical need to determine the prevalence of germline variants and the impact on clinical decision making in patients with BC. Thus, the aim of the current manuscript is to define the distribution of germline HRR variants in a European ancestry BC population and its clinical implications.

## 2 M&M

- This is an ongoing observational case–control study of data prospectively collected in patients who had received a diagnosis and a treatment for BC, who attended the outpatient follow‐up office of our tertiary high‐volume university hospital from September 2024 to February 2025 (last analysis). The study was founded by the Italian non‐profit charity AIRC (Fondazione AIRC per la Ricerca sul Cancro), by project IG 2020 ID 25027, and conducted in agreement with both the Declaration of Helsinki and the Italian laws and regulations. The protocol was subject to review and approval by the competent Independent Ethics Committee (ICH‐2812) and developed according to the IRB Guideline for Good Clinical Practice.
- Patients were included if they met all the following criteria: (i) age ≥ 18 years old; (ii) having received a diagnosis of BC regardless of the stage (nonmuscle invasive—NMIBC, or muscle‐invasive—MIBC; (iii) having undergone a diagnostic and at least a therapeutic procedure (TURB with or without adjuvant intravesical therapy according to EAU Guideline and/or Radical Cystectomy—RC) in our hospital in the last 5 years; (iv) having complete electronic chart records; (v) ECOG < 2; (vi) willing to be tested for germline variants. For the specific purposes of this survey, we excluded men with a previous or synchronous diagnosis of prostate cancer, upper urinary tract cancer, patients with recurrent low‐grade NMIBC enrolled in active surveillance programs,^11^ incomplete medical data, unwilling to consent, and any condition that in the judgement of the investigators would interfere with the subject's ability to provide informed consent, comply with study instructions, place the subject at increased risk, or which might confound the interpretation of study results.

Patients who accessed the BC follow‐up office, who met the inclusion and exclusion criteria and consented to participate, were asked to be tested for germline variants according to the previously published panel.^12^ In particular, we searched for germline variants in 20 DNA repair genes (gDRG) previously implicated in PCa (ATM, MLH1, ATR, MRE11A, BAP1, MSH2, BARD1, MSH6, BRCA1, NBN, BRCA2, PALB2, BRIP1, PMS2, CHEK2, RAD1C, FAM175A, RAD1D, GEN1 and XRCC1).^13^ More details can be found in the genetic analyses section. Variants were classified according to the American College of Medical Genetics and Genomics/Association for Molecular Pathology (ACMG/AMP) criteria^14^ into pathogenic (PV) (Class 5), likely pathogenic (LPV) (Class 4), or of uncertain significance (VUS) (Class 3), and ClinGen recommendations.

The primary endpoints were to report the rate of patients with BC having a PV/LPV/VUS and to identify the frequency of gDRG in the cohort. Furthermore, we compared the cohort with gHRR mutations with patients without variants. The two groups were matched for age at diagnosis, sex, ethnicity, stage and grade at diagnosis, pathological report, age at RC and clinical outcome.

Data were described as numbers and percentages, if categorical, or mean and standard deviation if continuous and approximately Gaussian, or median and range, otherwise. Adherence to the Gaussian distribution was verified with the Shapiro–Wilk test. The statistical analysis compared patients with and without the variants, matching them for age at diagnosis, sex, ethnicity, tumour stage and grade at diagnosis, pathological findings, age at radical cystectomy (RC) and clinical outcome.

## 3 GENETIC ANALYSES

Genomic DNA was isolated from 500 μL of a patient's peripheral blood sample using the Maxwell RSC Whole Blood DNA kit (Promega, Madison, WI, USA), according to the manufacturer's instructions. DNA was quantified on a Qubit fluorometer (Thermo Fisher Scientific, Waltham, MA, USA) using the Qubit dsDNA BR Assay kit (Thermo Fisher Scientific). Extracted DNA was sent to an external laboratory (Dante Labs, Tecnopolo D, L'Aquila) for library preparation and whole‐exome sequencing (WES). WES libraries were produced using the Agilent Sure Select exome v7 Library prep kit (Agilent Technologies, Santa Clara, CA, USA) and sequenced as 150 nt paired‐end reads on an Illumina NovaSeq6000 (Illumina, San Diego, CA, USA), according to standard procedure. Data were analysed using the Illumina DRAGEN Germline pipeline, which provides end‐to‐end (from .bcl to .vcf file) sequencing analysis. Reads were mapped against the GrCh37 human reference genome. Only variants in 20 DRGs included in the informed consent were extracted. Criteria for variant prioritization included: minimum variant coverage 30×, frequency in the gnomAD database lower than 1%, nonsynonymous variants (i.e., missense, nonsense/stopgain, frame‐shift, in‐frame coding indels and splicing). Clinvar classification, which was available, was considered, excluding benign/likely benign variants from the patient report. For missense variants, deleteriousness scores from CADD (>20) and REVEL (>0.5) were also considered to support variant pathogenicity. Class 3, 4 and 5 variants were reported to each patient in a detailed research report, which was discussed with an experienced medical geneticist for counselling. Class 4 and 5 variants were also confirmed in a diagnostic setting.

## 4 RESULTS

Of 137 patients who attended the BC follow‐up ambulatory from September 2024 to February 2025, 75 met all the inclusion and exclusion criteria; all of them were Caucasian (European ancestry). Most of the patients (*n* = 39) were excluded because they did not undergo the BC diagnosis or the primary treatment (TURBT/RC) at our hospital, and their complete medical history data were not available. Thirteen were rejected because of the fear of familial implication (they do not want to know their genetic germline profile after genetic consultation), and 10 did not report any reason to reject participation (The answer was ‘I am not interested in that’ or ‘anyway nothing would change’). Complete genetic data are currently available for 72 of the 75 enrolled patients.

The mean age at the diagnosis of BC was 65.5 years (SD 7.7); 66 were males and 9 females. Four patients reported a family history of BC, but none had a history of hereditary tumours in their family (e.g., Lynch syndrome). Sixty‐seven patients had a NMIBC (52 pTa and 15 pT1) and 8 had a MIBC; synchronous CIS was found in 10 patients (8 in NMIBC and 2 in MIBC). Thirty patients presented a low‐grade (LG) disease, and 45 had high‐grade (HG) BC. Fifteen patients presented a pathological non‐classical variant: squamous, glandular, sarcomatoid, micropapillary and neuroendocrine.

We found on average 140 variants per patient in the 20 analysed genes, of which only a few were rare (MAF < 1%) in the general population (average: 6 variants/patient, min 1, max 13). Twenty‐three patients (30.6%) presented with at least one PV, LPV or VUS variant (Table 1) in the following genes: ATM, ATR, BARD1, PMS2, BRCA2, CHEK2, MSH6, NBN, PALB2 and GEN1, for a total of 32 reported variants (Table 1). Within them, 7 (30.4%) patients had a double variant (CHEK2 + PMS2; ATM + MSH6; ATM + BARD1; ATR + BRCA2; ATM + ATR; ATM + ATR; CHEK2 + MSH6), and one (4.3%) patient had three variants (BARD1 + CHEK2 + XRCC2). The genes that most frequently carried LPV/VUS variants were the following: *ATM* (*N* = 6), *ATR* and *BARD1* (*N* = 4), *CHEK2* and *PMS2* (*N* = 3). Conversely, BRCA1 and 2 variants were observed only in three patients (one variant in *BRCA2* and two in *BRCA1*). Most of them were initially classified as VUS (*n* = 18), but recently, two of the VUS variants were re‐classified as PV and were associated with MIBC. Patients with two variants had a younger age (mean age 58.16, SD 3.02) and high‐grade BC; three of them were BCG unresponsive. Patients with a gDRG variant had a higher rate of histopathological variants, 34.8% versus 13.5%, while no difference was reported about concomitant CIS: 13% versus 13.5%, respectively. Comparing patients with NMIBC and MIBC, the frequency of gDRG variants was 25.8% (17/67) and 50% (4/8), respectively. Significantly, 9 patients (30%) over 30 with LG‐G1 disease presented with gDRG variants. Table 2 summarises all clinical data.

**TABLE 1:** gDRG variants identified in 72 bladder cancer patients.

Patient	Gene	Variant	Type	ACGM class	Sex	Age	BC type
DRG_142BC	CHEK2	p.D438Y	MISSENSE	VUS	M		
	PMS2	p.E77K	MISSENSE	VUS			
DRG_148BC	ATM	p.H1568R	MISSENSE	VUS	M		
	MSH6	R761G	MISSENSE	VUS			
DRG_151BC	ATM	p.P2974L	MISSENSE	**LPV**	M		
	BARD1	p.N118S	MISSENSE	VUS			
DRG_160BC	BARD1	p.S363T	MISSENSE	VUS	M		
DRG_168BC	ATM	p.K1992T	MISSENSE	VUS	M		
	ATR	p.E471Q	MISSENSE	VUS			
DRG_172BC	ATR	p.S902P	MISSENSE	VUS	M		
	BRCA2	p.G1376R	MISSENSE	VUS			
DRG_173BC	ATM	p.R1918T	MISSENSE	VUS	M		
	ATR	p.K764E	MISSENSE	VUS			
DRG_174BC	GEN1	p.T40Sfs*4	TRUNC	VUS	M		
DRG_176BC	PALB2	p.I676L	MISSENSE	VUS	M		
DRG_178BC	NBN	p.R215W	MISSENSE	VUS	M		
DRG_179BC	PMS2	p.G497D	MISSENSE	VUS	F		
DRG_180BC	BARD1	p.C639R	MISSENSE	VUS	M		
DRG_186BC	BRCA1	p.S1577Y	MISSENSE	VUS	M		
DRG_187BC	BRIP1	p.T260A	MISSENSE	VUS	F		
DRG_188BC	PMS2	p.A38V	MISSENSE	VUS	F		
DRG_195BC	GEN1	p.P367S	MISSENSE	VUS	F		
DRG_218BC	ATR	p.S1142G	MISSENSE	VUS	F		
DRG_221BC	ATM	p.F627C	MISSENSE	VUS	M		
DRG_223BC	CHEK2	p.D118E	MISSENSE	VUS	M		
	MSH6	p.I1115T	MISSENSE	**LPV**			
DRG_224BC	BARD1	p.P315L	MISSENSE	VUS	M		
	CHEK2	p.D134H	MISSENSE	VUS			
	XRCC2	p.V277Lfs*19	TRUNC	**LPV**			
DRG_226BC	PALB2	p.Y334C	MISSENSE	VUS	M		
DRG_230BC	ATM	p.R457L	MISSENSE	VUS	M		
DRG_232BC	BRCA1	A1669S	MISSENSE	VUS	F		

**TABLE 2:** Demographic and clinical data.

Variable	Without gDRG variant (*N* = 49)	With gDRG variant (*N* = 23)	*p*‐value
Mean age at diagnosis	66 ± 6.5	65.5 ± 3.5	NS
Stage	NMIBC: 48 Ta: 36 T1: 12 MIBC: 4	NMIBC: 19 Ta: 16 T1: 3 MIBC: 4	NS
Grade	LG: 21 HG: 31	LG: 9 HG: 14	NS
Cystectomy	6	4	NS
Indication to RC	MIBC: 4 BCG unresponsive: 2	MIBC: 4 BCG unresponsive: 1	NS
Age at RC	75.3 sd 4.1	60.5 sd 3.5	<0.005

Ten patients had an RC: 6 in the negative and 4 in the positive gDRG groups. In the negative gDRG group, 4 patients had MIBC, and 2 had very high‐risk BCG‐unresponsive NMIBC (confirmed at the definitive pathology). In the positive gDRG group, 3 patients had MIBC and 1 high‐risk BCG‐unresponsive NMIBC (confirmed at the definitive pathology). Patients with gHRR variants, who had received an RC, were significantly younger: 60 years versus 75 (*p* < 0.05). Table 1 reports this data.

## 5 DISCUSSION

In the current study, we describe the distribution of germline Class 3 to 5 DRG variants in a real‐life scenario and set a correlation with clinical situations.

Up to 4.3% of BC patients have a first‐degree relative with BC, and up to 50% of urothelial cancer patients have a family history of cancer.^2^ People who have family members with BC have a higher risk of getting it themselves. The increased incidence in these subjects may be caused by the fact that family members are exposed to the same cancer‐causing chemicals (like those in tobacco smoke or in environmental risk exposure). However, they may also share changes in some genes (like GSTM1 and NAT2) that make it hard for their bodies to break down certain toxins, which can make them more likely to get cancer.^15^ A small number of people inherit a gene syndrome that increases their risk for BC. A mutation of the retinoblastoma (RB1) gene can cause cancer of the eye in infants, but also increases the risk of BC.^16^ Cowden disease, caused by mutations in the PTEN gene, is linked mainly to cancers of the breast and thyroid, but people with this disease also have a higher risk of BC.^17^ Lynch syndrome (also known as hereditary non‐polyposis colorectal cancer, or HNPCC) is linked mainly to colon and endometrial cancer. People with this syndrome might also have an increased risk of BC, as well as other cancers of the urinary tract. Such evidence and recent studies argue that the association of BC with constitutional DNA changes might be of interest, as these clues may provide additional avenues for large molecular studies to identify the genetic components of shared heritability of elevated risk in BC families and new therapeutic opportunities.

The full pathogenic germline variant landscape and optimal genetic evaluation criteria in BC remain poorly understood. Other than a complex polygenic predisposition to urothelial and BC, a statistically significant proportion of cancer susceptibility, accounted for by genetic effects, has been highlighted: the Swedish Family‐Cancer Database identified a likely monogenic genetic predisposition to urinary BC in 7% of cases.^18^ Younger age at the time of diagnosis and affected relatives can be considered risk factors for both BC as well as non‐BC in families. It has been found that the close relatives of an affected proband diagnosed before 45 years of age have a risk of BC of about seven times as compared with controls. Germline class 4/5 (pathogenic or likely pathogenic variants) were found in 22% of patients with BC.^19^ Notably, 20% of variants were found in DNA damage repair genes, and 8.9% of variants were found in high‐penetrance genes. Oh, and co‐workers reported the genetic testing outcomes and clinic‐pathologic characteristics of patients with urothelial carcinoma (UC), which included upper urinary tract cancers.^7^ Among all tested patients, 35 (27%) had a confirmed pathogenic/pathogenic‐like variant (PV/LPV); 18 (14%) had a variant of unknown significance (VUS). The most frequent PV/LPV genes were MSH2 (*n* = 8, 6%), mainly for upper urinary tract cancer, BRCA2 (*n* = 5, 4%), CHEK2 (*n* = 4, 3%), APC (*n* = 4, 3%) and MUTYH (*n* = 4, 3%). Similarly, Lazzeri and colleagues, while conducting an enhanced PCa screening in healthy Caucasian men with germline DNA repair pathogenic variants, found two naïve patients with BC without prostate cancer.^20^

Recently, Pietzak et al. reported a large series of patients with NMIBC and germline variants. They found a high rate of P/LP germline variants in patients with high‐grade NMIBC (13.5%), which is a similar frequency to that among patients with locally advanced and metastatic urothelial cancer.^21^ In our study, the frequency of patients with NMIBC associated with at least one gDRG variant was 25.8%, significantly higher than reported by Pietzak. Furthermore, 9 patients (30%) over 30 with LG‐G1 disease presented with gHRR variants. Pietzak and colleagues did not find any mutation in patients with LG disease. The reason for such a difference might be due to the fact that they did not include VUS in their analysis, while we did. Previously, Contieri and colleagues reported that none of the patients were under AS for very low‐risk NMIBC PVs or VUS.^22^ In our analysis, patients under AS were ruled out.

Our study has strengths and weaknesses that must be considered. We investigated gHRR in a real‐life population who attend a dedicated single‐centre in‐house follow‐up, but limited to the European ancestry population. Second, we considered not only PV and PLV but also VUS. VUS might have a role beyond what is the present evidence (in our series, two of them were reclassified a PV), but their clinic‐pathological meaning remains unknown. Patients with a gHRR variant had a higher rate of pathological variants (34.8% vs. 13.5%), but no difference was reported in the frequency of CIS: 13% versus 13.5%, respectively. The small number of patients with pathological variants and CIS does not permit any strong conclusion. Finally, patients who have gHRR variants had a high risk of receiving RC at a younger age and being BCG unresponsive. This could be very attractive for risk stratification in patients with BC, but further data from larger series and international multicentric collaboration are mandatory. There are weaknesses as well. This is a monocentric study focused only on Caucasian white patients, with a short time follow‐up in a heterogeneous casuistic. Although we performed an exome analysis, we searched only for germline variants in 20 DNA repair genes (gDRG) previously implicated in PCa.^20^

## 6 CONCLUSION

Our data, in line with literature evidence, indicate that germline testing and counselling should be considered in any patient with BC, whether they are initially diagnosed with LG, HG, NMIBC, MIBC or metastatic disease. In addition to the characterisation of somatic alterations in tumour samples, the identification of germline pathogenic variants can provide additional information to guide informed and personalised therapeutic planning for patients and to enable risk‐based screening protocols for at‐risk relatives. So far, in BC disease, only a few associations between germline mutations and cancer risk have been thoroughly investigated. To achieve a wider use of both tumour genomic and germline genetic testing, an integrative approach led by scientific societies is necessary to involve physicians, patients and advocacy groups, to develop a shared strategy to advance the field and provide value‐based and reproducible standards of care for patients and their families. Inherited germline pathogenic, likely pathogenic (P/LP), and VUS variants could have profound implications for the development, treatment and screening of various cancer types. Identification of P/LP/VUS variants may be even more important, as most patients with gDRG could be informative cases for their relatives and activate screening in probands. Finally, our findings may also have therapeutic implications in BC. PARP inhibitors confer selective sensitivity against HRR‐deficient tumours and are a standard therapy in multiple tumour types for patients with deleterious germline mutations in BRCA1/2 and other DDR genes. In the future, they could also be considered for the treatment of BC.

## AUTHOR CONTRIBUTIONS

**R.H.:** Conceptualization; data analysis; supervision. **A.C.:** Conceptualization; data analysis; supervision. **G.L.:** Conceptualization; data analysis; project management. **N.B.:** Conceptualization; data analysis. **F.S.:** Writing—original draft; writing—review and editing. **A.F.:** Writing—original draft; writing—review and editing. **A.S.:** Conceptualization; data analysis. **M.P.:** Conceptualization; data analysis. **V.F.:** Conceptualization; data analysis. **P.C.:** Conceptualization; data analysis. **P.B.:** Conceptualization; data analysis. **A.B.:** Conceptualization; data analysis. **P.A.:** Conceptualization; data analysis. **R.A.:** Conceptualization; data analysis. **G.S.:** Conceptualization; data analysis. **P.C.:** Supervision. **M.L.:** Supervision; conceptualization; data analysis.

## CONFLICT OF INTEREST STATEMENT

None of the authors has any relevant disclosures, and none have any financial or non‐financial interests pertinent to the submitted work.

## Data Availability

Data from the Humanitas Research Hospital database will be made available upon request in compliance with our institution and IRB regulations.
